# Two-period linear mixed effects models to analyze clinical trials with run-in data when the primary outcome is continuous: Applications to Alzheimer's disease

**DOI:** 10.1016/j.trci.2019.07.007

**Published:** 2019-09-05

**Authors:** Guoqiao Wang, Andrew J. Aschenbrenner, Yan Li, Eric McDade, Lei Liu, Tammie L.S. Benzinger, Randall J. Bateman, John C. Morris, Jason J. Hassenstab, Chengjie Xiong

**Affiliations:** a Division of Biostatistics, Washington University School of Medicine, St. Louis, MO, USA; b Department of Neurology, Washington University School of Medicine, St. Louis, MO, USA; c Department of Radiology, Washington University School of Medicine, St. Louis, MO, USA; d Department of Psychological and Brain Sciences, Washington University School of Medicine, St. Louis, MO, USA

**Keywords:** Alzheimer's disease, Linear mixed effects model, Run-in clinical trials, Unequal randomization, Two-period models

## Abstract

**Introduction:**

Study outcomes can be measured repeatedly based on the clinical trial protocol before randomization during what is known as the “run-in” period. However, it has not been established how best to incorporate run-in data into the primary analysis of the trial.

**Methods:**

We proposed two-period (run-in period and randomization period) linear mixed effects models to simultaneously model the run-in data and the postrandomization data.

**Results:**

Compared with the traditional models, the two-period linear mixed effects models can increase the power up to 15% and yield similar power for both unequal randomization and equal randomization.

**Discussion:**

Given that analysis of run-in data using the two-period linear mixed effects models allows more participants (unequal randomization) to be on the active treatment with similar power to that of the equal-randomization trials, it may reduce the dropout by assigning more participants to the active treatment and thus improve the efficiency of AD clinical trials.

## 1 Introduction

To facilitate the development of disease-modifying therapies for Alzheimer's disease (AD), trial-ready cohorts have been established where participants provide longitudinal measurements on clinical, cognitive, or other measures while investigational drugs are being identified [1], [2]. In this prerandomization period, the primary end points for the future clinical trials, such as clinical or cognitive tests, are assessed based on the master protocol of the platform trials allowing for easy incorporation of the prerandomization data into the primary analysis. This longitudinal period before randomization is historically referred to as the run-in period during which potential participants who have met all entry criteria for a randomized clinical trial are assigned no regiment or the same regimen (e.g., placebo) [3]. Planning a run-in period before randomization has been extensively implemented in many landmark clinical trials [4], [5], [6], [7] including trials for AD [7], and it is expected to continue to be an essential design element [8]. The run-in design has been implemented in the dominantly inherited Alzheimer network (DIAN) trial unit platform trial [1] and the European Prevention of Alzheimer's Dementia Proof of Concept Platform [2]. In these settings, each participant's duration and the number of primary end point assessments in the run-in period may vary and depend on the timing of enrollment.

The assessments of the primary outcome collected during run-in can potentially be used in the primary efficacy analysis at the end of the clinical trials. However, it has not been fully established how best to incorporate run-in data into final analyses. When only a single assessment is collected in the run-in period, the run-in data are often used as a covariate in the primary analysis model [9], whereas when multiple assessments are available, the rate of change (slope) in the run-in period can be used as a covariate [10] within linear mixed effects (LMEs) models or mixed effects models for repeated measures frameworks. Although these methods are helpful, they did not fully take advantage of the run-in data especially when multiple run-in assessments are present. In addition, when the run-in duration varies by individual, the variability of the run-in data over time is not fully accounted for. In AD clinical trials, the primary end points are continuous and the primary efficacy inference is based on the slowing of the rate of decline in cognition. For these types of end points, we propose a two-period (run-in period and randomization period) LME model to simultaneously model the run-in data and the randomization data. We investigated the behavior of the two-period LME by simulating clinical trials using parameters estimated from the DIAN study and evaluated the gain in power compared with the LME models using run-in data (baseline or rate of change) as a covariate.

The remainder of this article is as follows. Section 2 presents the model formulations of the LME with a covariate and the two-period LME. Section 3 evaluates model behavior through simulated hypothetical clinical trials. Section 4 presents the power formulas, and Section 5 presents the discussion.

## 2 Methods

### 2.1 Using information from run-in period as a covariate

As mentioned, the traditional model to analyze clinical trials with run-in data is LME model. The baseline assessment or the rate of change estimated using the run-in assessments will be included in the LME as a covariate. This traditional model can be expressed as follows.

Let *y*_*ijk*_ denote the longitudinal assessments for subject *i* at time *t*_*ij*_ for treatment group *k*, and it can be modeled as

(1) $$y_{ijk}=\mu_{0}+u_{0i}+\beta_{1}*X_{1i}+\beta_{2}*X_{1i}*t_{ij}+\left(\mu_{1k}+u_{1i}\right)*t_{ij}+\varepsilon_{ij}$$

where *u*_0*i*_, *u*_1*i*_ are the random effects for the intercept and the slope and follow a bivariate normal distribution

$$\left(\begin{array}{c} u_{0i}\\ u_{1i} \end{array}\right)\sim N(0,\;\;\left[\begin{array}{cc} \sigma_{u_{0i}}^{2} & \sigma_{u_{0i}u_{1i}}\\ \sigma_{u_{0i}u_{1i}} & \sigma_{u_{1i}}^{2} \end{array}\right]);$$

the residual follows normal distributions $\varepsilon_{ij}\sim N\left(0,\;\sigma_{e}^{2}\right)$, β's are the coefficients associated with the corresponding covariate *X*_1*i*_, μ_0_ is the baseline group mean and is assumed to be the same for the treatment group and the placebo group because of randomization, μ_1*k*_ represents the rate of change, *i*=1, 2, …, *n*, *j*=0, 1, …, *n*_*i*_, and *k*=1, 2 represents the placebo group and the treatment group. The primary efficacy test is to compare the rate of change of the treatment group (μ_12_) to that of the placebo group (μ_11_) during the randomization period.

### 2.2 Two-period LME

We propose the two-period LME to model the run-in period and the randomization period simultaneously. We investigate two scenarios: the slope of the placebo group in the run-in period is the same as (scenario 1) or is different from (scenario 2) that in the postrandomization period of the placebo group.

#### 2.2.1 Scenario 1

When the slopes are the same, the two-period LME model can be presented as

(2) $$y_{ijk}=\{\begin{array}{cc} \mu_{0}+u_{0i}+\left(\mu_{1}+u_{1i}\;\right)*t_{ij}+\varepsilon_{ij}, & t_{ij}\leq t_{ibl}\\ \mu_{0}+u_{0i}+\left(\mu_{1}+u_{1i}\right)*t_{ibl}+\left(\mu_{1}+\Delta \mu_{k}+u_{1i}\;\right)*{\left(t_{ij}-t_{ibl}\right)}_{+}+\varepsilon_{ij},\; & t_{ij}>t_{ibl} \end{array}\text{,}$$

where *Δ*μ_*k*_ represents the treatment effect and equals to 0 for the placebo group; *t*_*i*bl_ represents the baseline time of the randomization period; (*t*_*ij*_− *t*_*ibl*_)_+_ = max (*t*_*ij*_ − *t*_*ibl*_, 0); *j* = 0, 1, …, *bl, bl* + 1, *bl* + 2, *bl* + 3,…; *t*_*i*0_ = 0 represents the baseline of the run-in period; *μ*_0_, *u*_0*i*_, *u*_1*i*_, and ε_*ij*_ are defined in the same way as in Section 2.1; *μ*_1_ is the slope of the placebo group in the run-in period and the randomization period.

#### 2.2.2 Scenario 2

Similarly, when the slopes are different, the two-period LME model can be presented as

(3) $$y_{ijk}=\{\begin{array}{c} \mu_{0}+u_{0i}+\left(\mu_{1}+u_{1i}\right)*t_{ij}+\varepsilon_{ij}\text{, }\;t_{ij}\leq t_{ibl}\\ \mu_{0}+u_{0i}+\left(\mu_{1}\;+u_{1i}\right)*t_{ibl}+\left(\mu_{2}+\Delta \mu_{k}+u_{2i}\right)*{\left(t_{ij}-t_{ibl}\right)}_{+}+\varepsilon_{ij},\;\;t_{ij}>t_{ibl} \end{array},$$

where *μ*_1_ and *μ*_2_ are the slopes of the placebo arm during the run-in period and the randomization period; *Δμ*_*k*_, (*t*_*ij*_ − *t*_*ibl*_)_+_, and *t*_*ibl*_ are defined as in equation (2); *μ*_0_ and *ε*_*ij*_ are defined in the same way as in Section 2.1, whereas *u*_0*i*_, *u*_1*i*_, and *u*_2*i*_ follow a multivariate normal distribution:

$$\left(\begin{array}{c} \begin{array}{c} u_{0i}\\ u_{1i} \end{array}\\ u_{2i} \end{array}\right)\sim N\left(\begin{array}{c} 0\\ 0\\ 0 \end{array},\;\;\;\left[\begin{array}{ccc} \sigma_{u_{0i}}^{2} & \sigma_{u_{0i}u_{1i}} & \sigma_{u_{0i}u_{2i}}\\ \sigma_{u_{0i}u_{1i}} & \sigma_{u_{1i}}^{2} & \sigma_{u_{1i}u_{2i}}\\ \sigma_{u_{0i}u_{2i}} & \sigma_{u_{1i}u_{2i}} & \sigma_{u_{2i}}^{2} \end{array}\right]\right).$$

The duration of the run-in period could be different for each individual, and there can be multiple assessments during the run-in period.

## 3 Evaluation of the behavior of various LMEs

### 3.1 Participants from DIAN study

The DIAN study is an international, longitudinal observational study established in 2008. As of June 2018 it has enrolled 529 participants from families with confirmation of a causal autosomal dominant Alzheimer's disease mutation and a 50% chance of inheriting the mutation. The details of participants' demographics, clinical, cognitive, imaging, and biochemical measures have been reported in previous publications [11], [12]. For this study, only mutation carriers were included because mutation noncarriers are healthy control subjects and are not allowed to be given any treatment. The data include DIAN quality-controlled data from July 2008 to June 2018 consisting of 310 mutation carriers. As many clinical trials use a cognitive composite score as the primary outcome [1], [13], we formed a cognitive composite consisting of a digit symbol substitution task test from the Wechsler Adult Intelligence Scale-Revised [14], the Mini-Mental State Examination [15], the DIAN word list delayed recall test [16], and the Wechsler Memory Scale-Revised logical memory delayed recall test [17]. The cognitive composite is an average of the *z*-score of these four tests [11], [12].

### 3.2 Power comparison

We first estimated the baseline mean (μ_0_), the annual slope (μ_1_), and the variance-covariance for the random intercept and the random slope

$$\left[\begin{array}{cc} \sigma_{u_{0i}}^{2} & \sigma_{u_{0i}u_{1i}}\\ \sigma_{u_{0i}u_{1i}} & \sigma_{u_{1i}}^{2} \end{array}\right]$$

and the residual $\sigma_{e}^{2}$. Furthermore, we assume *μ*_2_ = 0.9∗μ_1_, $\sigma_{u_{2i}}^{2}=0.9^{2}\sigma_{u_{1i}}^{2}$, the correlation between *u*_0*i*_ and *u*_2*i*_ is 0.4, and between *u*_1*i*_ and *u*_2*i*_ is 0.8. The values of these variables are presented in Table 1.

**Table 1:** Estimated simulation parameters for the cognitive composite using the DIAN observational study

Parameter	Variance-covariance matrix			Mean
	*u*_0*i*_	*u*_1*i*_	*u*_2*i*_	
*u*_0*i*_	1.0656	0.09253	0.05674	−0.6289
*u*_1*i*_	0.09253	0.02331	0.01678	−0.09506
*u*_2*i*_	0.05674	0.01678	0.01888	−0.08555
$\sigma_{e}^{2}$	0.05160			

Abbreviation: DIAN, dominantly inherited Alzheimer network.

**Fig. 1:**
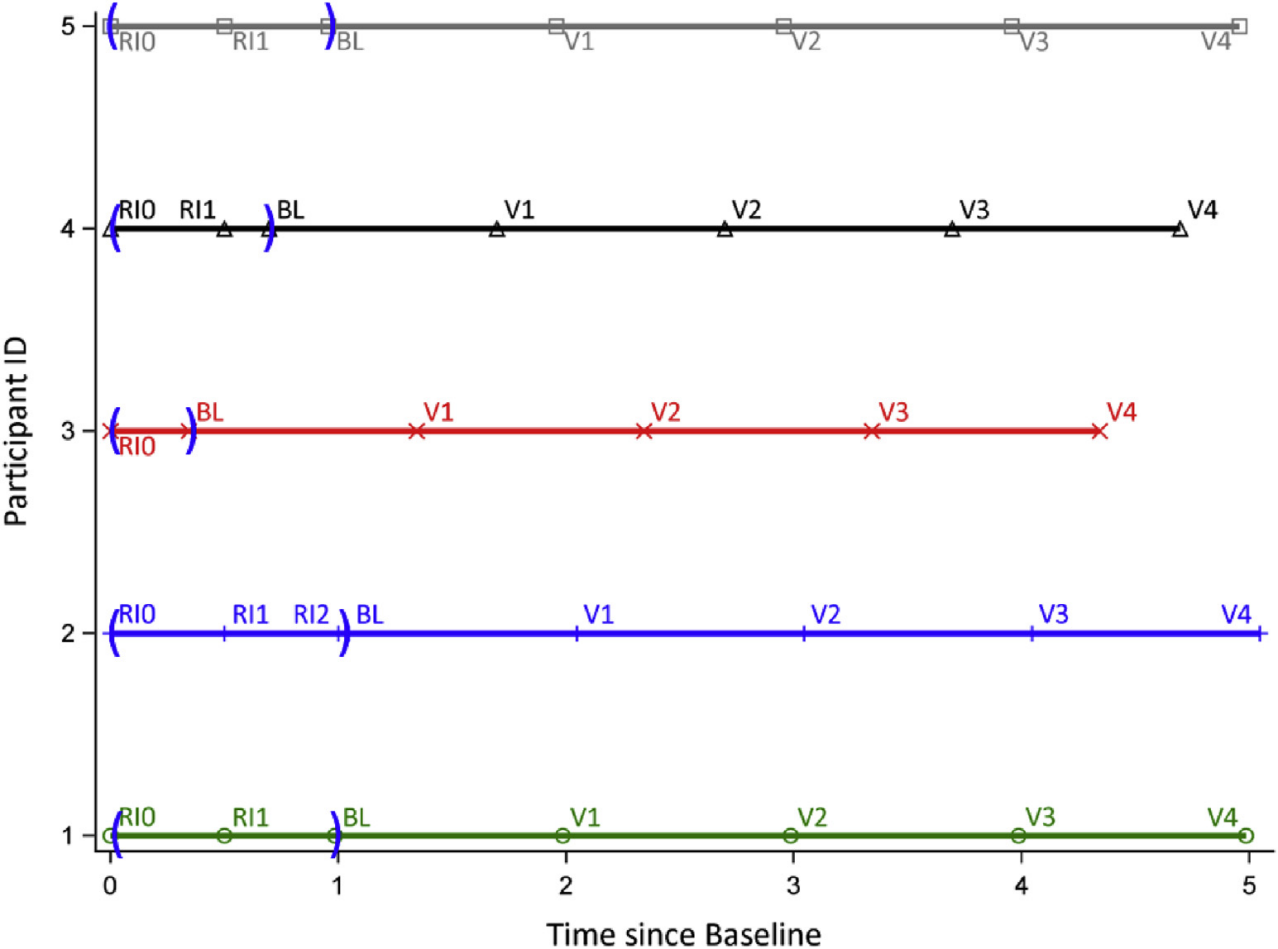
The run-in period and the randomization period. The run-in period was simulated using a uniform distribution (0.3, 1.2). The “BL” assessments of the randomization period were measured at the time of randomization and could be very close to the last run-in assessments (participant 2). The run-in period had at least one (participant 3) and up to three (participant 2) assessments. Abbreviation: BL, baseline.
• Individual duration of the run-in period: uniform distribution (0.3, 1.2) (Fig. 1).
• Primary outcome measured every 0.5 year in the run-in period until the individual was randomized to the treatment, and then every 1 year in the randomization period.
• The last measurement in the run-in period is also the first one in the randomization period, and it was measured at the time of randomization regardless how far this measurement was from the last measurement in the run-in period (Fig. 1).
• The slopes of the placebo group in the run-in period and the randomization period were the same and the primary outcome was simulated based on formula (2).
• The slopes of the placebo group in the run-in period and the randomization period were different and the primary outcome was simulated based on formula (3).
• Effect size (% reduction in the slope): 0%, 30%, 40%, 50%, and 60%.

To evaluate the advantage of the two-period model relative to the traditional LME with/without run-in data as a covariate, we simulated clinical trials based on data of the DIAN study to closely mimic AD trials. This creates four models for comparison: (1) traditional LME without run-in, (2) traditional LME with the first run-in assessment as a covariate, (3) traditional LME with the slope of change across all run-in visits included as a covariate, and (4) the two-period model with run-in. Simulation SAS codes are provided in the Supplementary Material. We simulated trials with 1:1 and 3:1 treatment to placebo randomization ratio for a total 400 patients. Overall, we make the following assumptions for our simulated trials:

• Four-year trial after randomization without/with run-in period (Fig. 1).

**Fig. 2:**
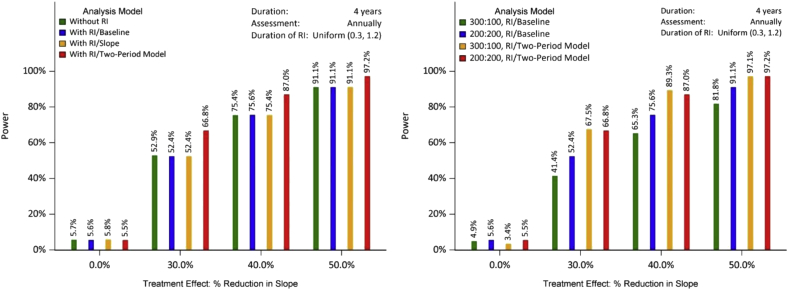
Power/type I error for each design (with/without RI), different analysis models, and different randomization ratios assuming the same rate of change in the RI period and the randomization period. Sample size for the left panel: 200/arm. With RI/slope: with RI, LME with individual slope as a covariate; with RI/baseline: with RI, LME with individual baseline value as a covariate. 300:100 RI/baseline: 300 on treatment and 100 on placebo; 200:200:200 on treatment and 200 on placebo. Abbreviations: LME, linear mixed effect; RI, run-in.

**Fig. 3:**
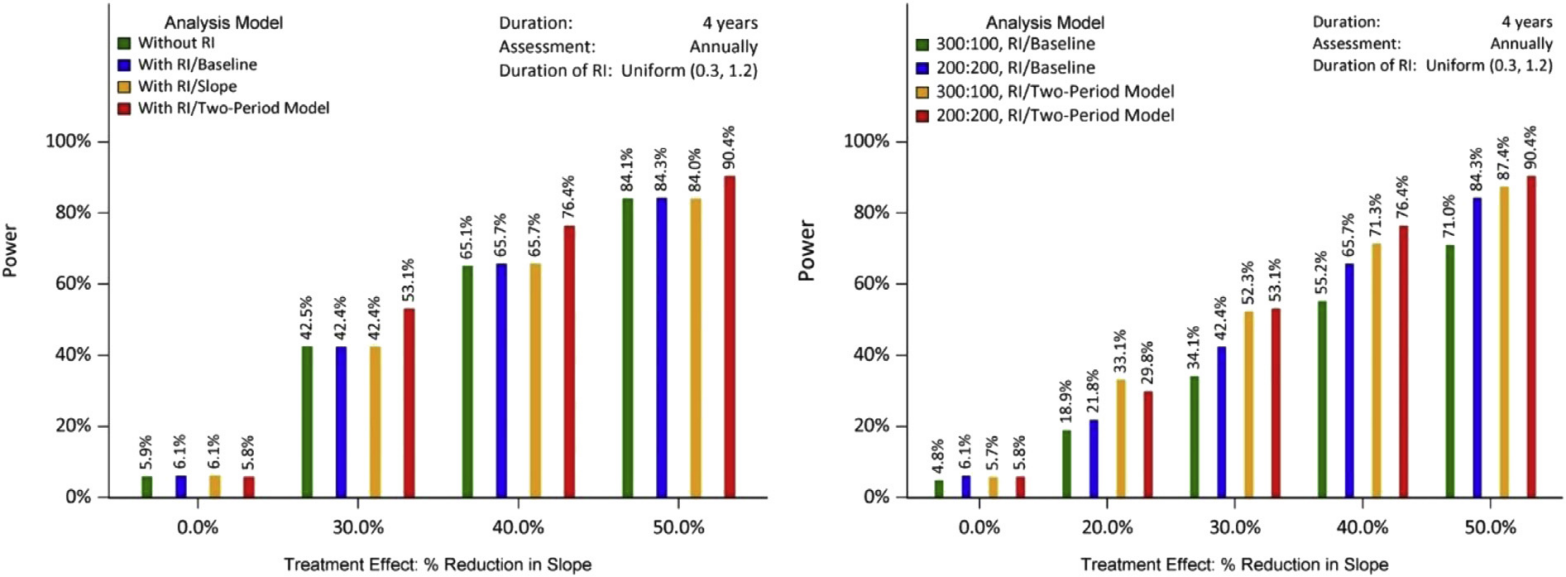
Power/type I error for each design (with/without RI), different analysis models, and different randomization ratios assuming the different rates of change in the RI period and the randomization period. Sample size for the left panel: 200/arm. With RI/slope: with RI, LME with individual slope as a covariate; with RI/baseline: with RI, LME with individual baseline value as a covariate. 300:100 RI/baseline: 300 on treatment and 100 on placebo; 200:200:200 on treatment and 200 on placebo. Abbreviations: LME, linear mixed effect; RI, run-in.

For each of the models mentioned previously, we simulated 1000 clinical trials, and calculated type I error and power as the proportion of 1000 simulated trials per scenario with *P* values less than .05. The 4-year trials without run-in were used as the anchor point to demonstrate the power improvement of run-in trials. The power/type I error comparison is presented in Figs. 2 and 3. Each figure includes the comparison among the four types of design/models with 1:1 randomization (left panel) and the comparison between the 1:1 randomization and the 3:1 randomization (right panel). Fig. 2 represents the scenario where the slope of the placebo group in the run-in period is the same as that in the randomization period, whereas Fig. 3 displays the case where the two slopes are different. For both scenarios, the type I error is well controlled for all models. The two-period LME leads up to 15% increase in power for the same slope scenario with 1:1 randomization. When comparing the 3:1 with the 1:1 randomization, the two-period LME yields almost identical power, whereas the traditional LME yields more power for the equal randomization. For the two-slope scenario, the power improvement for the two-period model is up to 11% compared with the LME with a covariate. The 3:1 randomization has slightly less power than 1:1, but the discrepancy for two-period LME is much smaller than that for the traditional LME.

## 4 Power estimation of the two-period LME

Under the framework of LME, we first presented the power estimation formulas for the two-period model assuming no dropout and no intermittent missing data, then proposed the algorithm to account for the dropout.

### 4.1 The same slope for the placebo group in the run-in period and the randomization period

To get a closed formula, we rewrote the treatment group of equation (2) as

$$y_{ijk}=\{\begin{array}{cc} \mu_{0}+u_{0i}+\left(\mu_{1}+u_{1i}\;\right)*t_{ij}+\varepsilon_{ij}\text{, }\; & t_{ij}\leq t_{ibl}\\ \mu_{0}+u_{0i}+\left(\mu_{1}+u_{1i}\;\right)*t_{ij}+\Delta \mu_{k}*{\left(t_{ij}-t_{ibl}\right)}_{+}+\varepsilon_{ij},\; & t_{ij}>t_{ibl} \end{array}$$

Further simplification yielded

$$y_{ij}=\mu_{0}+u_{0i}+\left(\mu_{1}+u_{1i}\;\right)*t_{ij}+\Delta \mu_{k}*{\left(t_{ij}-t_{ibl}\right)}_{+}+\varepsilon_{ij}.$$

The null hypothesis is *H*_0_: *Δ*μ_2_=0 and the alternative is *H*_1_:  *Δ*μ_2_≠0. For the fixed effects, the design matrix (***X***) of the treatment group is

$$X=\left(\begin{array}{ccc} 1 & t_{i0} & 0\\ 1 & t_{i1} & 0\\ \vdots & \vdots & \vdots \\ 1 & t_{ibl} & 0\\ 1 & t_{ij} & t_{ij}-t_{ibl}\\ 1 & t_{ij+1} & t_{ij+1}-t_{ibl}\\ \vdots & \vdots & \vdots \\ 1 & t_{in_{i}} & t_{in_{i}}-t_{ibl} \end{array}\right),$$

although it only includes the first two columns for the placebo group. The design matrix for the random effect also includes only the first two columns. Thus *E* (***Y***_***i***_|***U***_***i***_) = ***X*β** + ***ZU***_***i***_, where $\beta =\left(\begin{array}{c} \mu_{0}\\ \mu_{1}\\ \Delta \mu_{2} \end{array}\right)$ represents fixed effects, $U_{i}=\left(\begin{array}{c} u_{0i}\\ u_{1i} \end{array}\right)$ represents the random effects, ***U***_***i***_~***N*** (0,***G***). The fixed effect can be estimated by: βˆ = (*X*^T^Σ^−1^*X*)^−1^ *X*^T^Σ^−1^*Y*, and V (βˆ) = (*X*^T^Σ^−1^*X*)^−1^, where **Σ** = ***R*** + ***ZGZ***^***'***^, ***R*** is the diagonal residual matrix. To determine the power for a complex run-in design, we adopted the same strategy as in a previous study [10]. This is to calculate the variance/standard deviation (*s*) for a single subject and then estimate the standard error for a given sample size. Briefly, first, using pilot data or published results, we estimated the residual variance *R* and the covariance of the random intercepts and random slopes. Then plugging the design matrix ***X*** and ***Z*** for a single subject into **Σ** and V(βˆ) sequentially to estimate *s* for *Δ*μ. Next, the power for a trial with *N*_T_ subjects in the treatment group and *N*_P_ subjects in the placebo group can be determined from

$$1-\gamma =\mathrm{Pr}\left(\left|\frac{\Delta \mu_{2}}{s/\sqrt{N_{T}}}\right|\geq z_{\alpha /2}|H_{1}:\;\;\mu_{2}=\delta \right)=\mathrm{Pr}\left(Z\geq z_{\alpha /2}-\frac{\delta }{s/\sqrt{N_{T}}}\right)+\mathrm{Pr}\left(Z\leq -z_{\alpha /2}-\frac{\delta }{s/\sqrt{N_{T}}}\right)\text{,}$$

where α is the type I error and is often set to be 5% and γ is the type II error and is often set to be 20%; *z*_α_ is upper αth quantile of the standard normal distribution.

It is noted that the variance of *Δ*μ_2_ is estimated using all the data from the *N*_T_+*N*_P_ subjects, but the standard error (*s*√*N*_T_) is only related to *N*_T_. Thus, theoretically, given the total sample size, the larger the *N*_T_, the more power the run-in design has, leading to more power for the unequal randomization than the equal randomization. This benefit is attributed to two facts: (1) the same slope for the placebo group in both periods; and (2) the run-in data help estimate the slope of the placebo group and the variances of the random effects and the residuals.

### 4.2 Different slopes for the placebo group in the run-in period and the randomization period

In this scenario, we rewrote equation (3) as

$$y_{ijk}=\{\begin{array}{c} \mu_{0}+u_{0i}+\left(\mu_{1}\;+\;\;u_{1i}\;\right)*t_{ij}+\varepsilon_{ij},\;\;\;\;\;\;\;\;\;\;\;\;\;\;\;\;\;\;\;\;\;\;\;\;\;\;\;\;\;\;\;\;\;\;\;\;\;\;\;\;\;\;\;\;\;\;\;\;\;\;t_{ij}\leq t_{ibl}\\ \mu_{0}+u_{0i}+\left(\mu_{1}\;+\;\;u_{1i}\;\right)*t_{ibl}+\left(\mu_{2k}+\;\;u_{2i}\;\right)*{\left(t_{ij}-t_{ibl}\right)}_{+}+\varepsilon_{ij},\;\;\;\;\;\;\;\;t_{ij}>t_{ibl} \end{array}\text{,}$$

where *μ*_2*k*_ = *μ*_2_+*Δ*μ_*k*_, *k*=1, 2 represent the placebo group and the treatment group. The null hypothesis is *H*_0_: *μ*_21_ − *μ*_22_ = 0 and the alternative is *H*_1_: μ_21_ − μ_22_≠0. Then the design matrices for the fixed effects and the random effects for formula (3) are the same, and they are also the same for both groups:

$$X=\left(\begin{array}{ccc} 1 & t_{i0} & 0\\ 1 & t_{i1} & 0\\ \vdots & \vdots & \vdots \\ 1 & t_{ibl} & 0\\ 1 & t_{ibl} & t_{ij}-t_{ibl}\\ 1 & t_{ibl} & t_{ij+1}-t_{ibl}\\ \vdots & \vdots & \vdots \\ 1 & t_{ibl} & t_{in_{i}}-t_{ibl} \end{array}\right);$$

$$\beta_{k}=(\begin{array}{c} \mu_{0}\\ \mu_{1}\\ \mu_{2k} \end{array}),\;\text{and}\;U_{i}=\left(\begin{array}{c} u_{0i}\\ u_{1i}\\ u_{2i} \end{array}\right)\text{.}$$

Like Section 4.1, V (βˆk) can be obtained for a single subject using the aforementioned formulas for **Σ** and for V (βˆ). The power for a total sample size of *N*_T_ + *N*_P_ can be estimated from

$$1-\gamma =\mathrm{Pr}\left(\left|\frac{\mu_{21}-\mu_{22}}{s\sqrt{\left(1/N_{P}\right)+\left(1/N_{T}\right)}}\right|\geq z_{\alpha /2}|H_{1}:\;\mu_{21}-\mu_{22}=\delta \right)=\mathrm{Pr}\left(Z\geq z_{\alpha /2}-\frac{\delta }{s\sqrt{\left(1/N_{P}\right)+\left(1/N_{T}\right)}}\right)+\mathrm{Pr}\left(Z\leq -z_{\alpha /2}-\frac{\delta }{s\sqrt{\left(1/N_{P}\right)+\left(1/N_{T}\right)}}\right)\text{,}$$

where α, γ, and *z*_α_ are defined as in Section 4.2.

### 4.3 Algorithm to account for dropout

For scenarios with dropout, the sample size in the power formulas can be approximated by $N_{\text{dropout}}=\left(N_{\text{no}-\text{dropout}}/{\left(1-m\right)}^{n}\right)$, where *m* is the annual dropout rate, *n* is the total duration in years, *N*_dropout_ and *N*_no-dropout_ are the sample sizes for each treatment group with/without dropout. This method assumes that participants who drop out before the end of study do not contribute to the estimate of the treatment effect and its variance at all, and thus will underestimate the power and overestimate the sample size. An alternative method that accounts for the contribution of the early dropout participants has been proposed in previous research [10], [18]. Briefly, assuming the proportion and the sample size for each dropout pattern are *p*_*i*_ and *n*_*i*_ for a given treatment group, then the total sample size for that treatment group is approximated by [10], [18]

$$N=\frac{1}{\left(p_{1}/n_{1}+\cdots +p_{k}/n_{k}\right)},$$

where *k* is the total number of dropout patterns for this given treatment group. This method, however, assumes no intermittent missing data within each dropout pattern, or data after the intermittent missing data do not contribute.

## 5 Discussion

In this article, we proposed the two-period LME model to analyze clinical trials with run-in design when the efficacy inference is based on the rate of change. This two-period LME model offers two important benefits when compared with a traditional LME that uses measures from run-in as covariates: (1) model the run-in data directly instead of using them as covariates; and (2) assign more participants to the active treatment without losing power compared with the traditional equal randomization clinical trials because of the fact that the run-in data serve as placebos. The first advantage allows the luxury to fully account for the run-in information in terms of the number and the frequency of assessments, and yields more accurate estimation of the variance-covariance matrix of the random effects and the within-subject error. The latter may greatly appeal to participants to enroll and remain the trials and maintain drug compliance (as they are more likely to be assigned to the treatment arm), which is especially important for diseases without any effective treatments such as AD. Furthermore, we also provided concise power estimation formulas for the two-period LME model by manipulating the design matrices of the fixed effects and the random effects. Similar manipulation of the design matrices will generalize the two-period model to other variation of run-in designs such as all participants in the run-in period are given the active treatment.

The proposed two-period model is very flexible, in that it allows the fixed effects (slopes), the random effects, and even the ancillary parameters to be different in the two periods. The flexibility can alleviate various concerns about the run-in design. For example, assuming the slope in the run-in period to be different from that in the randomization period takes care of the concern that participants may behave differently before and after randomization. Using the parameters estimated from the DIAN study, we conducted extensive simulations to evaluate the model behavior mimicking real AD clinical trials. Also we showed that the two-period LME model yielded accurate estimations of the treatment effect, controlled type I error, and led to large increases in power compared with models that used the run-in data as covariates. An additional advantage of the two-period LME is that it can be implemented using the well-established SAS procedures such as PROC NLMIXED (see Supplementary Material for details), which makes these models easier to use.

It is important to note that our focus is to propose an optimal model for analysis of run-in clinical trials, it was not our intent to compare trials with and without run-in design although we anchored the comparison based on the trials without run-in. For such comparison, extensive research has been done by Frost et al. [10]. Under the framework of LME and using three data points (one run-in assessment, baseline assessment, and one postrandomization assessment), Frost et al. demonstrated that given the same follow-up duration the run-in designs can be more efficient (requiring smaller sample size) than designs without run-in provided that true between-subject variability in the rate of change (slopes) is large relative to within-subject error [10]. Our study was inspired by theirs, but different in that the two-period LME is more general, and its power calculation formula can handle any number of assessments and any assessment schedule both in the run-in period and the randomization period. Because both studies are under the same framework, the conclusions of Frost et al. also apply to the two-period LME model. For AD clinical trials, the primary outcome is usually a cognitive test [19], [20], [21] or a composite of multiple cognitive tests [1], [13]. For these cognitive outcomes, the between-subject variability in the rate of change (slopes) is typically smaller relative to within-subject error, thus given the same follow-up duration and the same sample size, trials without the run-in design should have larger power than those with run-in because the former put participants on the treatment from the beginning and the latter after the run-in period. Of course, it is always optimal to start participants on a treatment as soon as possible. In other words, a 4-year AD trial with 1 year of run-in (in which treatment only begins after the first year) is always less powerful/optimal than a 5-year AD trial without run-in (in which treatment begins from the baseline). However, our results show that if run-in data are available (e.g., from a prior observational study) or if some cognitive data can be collected when other aspects of the clinical trial are still being developed (e.g., when a drug is being finalized) then the two-period model provides an optimal way to combine run-in data with trial data to maximize the probability of detecting a significant treatment effect.

Our study has some limitations. First, the two-period LME assumes the rate of change during the follow-up is linear. Although multiple studies have shown that the decline in cognition was linear, especially within a relatively short period like 2 years [22], [23], it is not clear if this linearity assumption is still true over a longer course of follow-up or under the influence of disease-modifying treatments. Second, although some clinical trials with run-in designs have been conducted, we were not able to obtain these real clinical trial data to validate the two-period LME model. Instead, we simulated clinical trials using parameters estimated from a longitudinal observational AD study to mimic real clinical trials as closely as possible.

In summary, the two-period LME model optimizes the use of run-in data, is flexible to account for design variations, can increase the power of clinical trials, and allows more participants (unequal randomization) to be on the active treatment without losing power compared with the equal-randomization trials. It may serve as a superior primary analysis model for platform clinical trials where “trial-ready” populations are enrolled in longitudinal observational studies waiting for randomization to clinical trials such as DIAN and European Prevention of Alzheimer's Dementia.

**Research in context**

1. Systematic review: We reviewed the existing literature about statistical models that can be used to analyze clinical trials with run-in design. Most methods use the run-in data as a covariate, leading to inefficient use of the run-in data.
2. Interpretation: The proposed two-period linear mixed effects models jointly model the run-in data and the double-blinded randomized data, can lead up to 15% power increase, and allow unequal randomization without losing significant power compared with equal randomization.
3. Future directions: The generalization of the two-period models to other mixed effects model such as the mixed effects model for repeat measures using time as categorical is of great interest as mixed effects model for repeat measures does not have the linearity assumption.
