# Supplementary material for: Two-period linear mixed effects models to analyze clinical trials with run-in data when the primary outcome is continuous: Applications to Alzheimer's disease
Source: Alzheimers Dement (N Y). 2019 Sep 5;5:450–7. doi: 10.1016/j.trci.2019.07.007 (PMC6732759; doi:10.1016/j.trci.2019.07.007)
Supplement: Supplementary Material [file mmc1.pdf]

Supplemental materials for manuscript: Two-period linear mixed effects models to analyze clinical trials with run-in data when the primary outcome is continuous: applications to Alzheimer disease, by Guoqiao Wang et. al.

***=====================Scenario 1==**

```
/*===========================================~~~~~~~~~~~~~~~~~~~~~~~~~
~~
Simulation based on Andy's data
Two slopes: before Randomization: estimated placebo slope:-0.1124
After randomization: 90%;
Correlation between slopes: 0.8
Correlation between int and 2nd slope: 0.4
Assessments: every 0.5 y in RI period, annually in randomization
period
Maximum of 3 assessments in the RI period: 0, 0.5, 1.0
===========================================~~~~~~~~~~~~~~~~~~~~~~~~~~~
~~*/

data scov_pla(type=COV) ;
   input _TYPE_ $ 1-4 _NAME_ $ 9-10 S1 S2;
   datalines ;
COV     S1      1.0656 0.09253
COV     S2      0.09253 0.02331
MEAN            -0.6289 0
;run;

%macro
CRI(sst,ssp,slope1,uni_lower,uni_upper,error_sd,theta,theta_int,iterat
ion);
%do ite=1 %to &iteration;
*================Pla====================;
proc simnorm data=scov_pla outsim=pla numreal = &ssp; var s1-s2;run;
data pla;set pla;group='P';rename  s1=int s2=ind_rc1;run;
data pla;set pla;bl_time=rand('uniform',&uni_lower,&uni_upper);run;
*generate placebo data: 4-years after run-in;
data p_wide;set pla;id=_n_;
z_0=int+rand('normal',0,&error_sd);
if 0.5<bl_time then
z_01=int+(&slope1+ind_rc1)*0.5+rand('normal',0,&error_sd);
      else z_01=.;
if 1.0<bl_time then
z_02=int+(&slope1+ind_rc1)*1.0+rand('normal',0,&error_sd);
      else z_02=.;
```

```
z_bl=int+(&slope1+ind_rc1)*bl_time+rand('normal',0,&error_sd);
z_1=int+(&slope1+ind_rc1)*bl_time+1*(&slope1+ind_rc1)+rand('normal',0,
&error_sd);
z_2=int+(&slope1+ind_rc1)*bl_time+2*(&slope1+ind_rc1)+rand('normal',0,
&error_sd);
z_3=int+(&slope1+ind_rc1)*bl_time+3*(&slope1+ind_rc1)+rand('normal',0,
&error_sd);
z_4=int+(&slope1+ind_rc1)*bl_time+4*(&slope1+ind_rc1)+rand('normal',0,
&error_sd);
run;
proc simnorm data=scov_pla outsim=tx numreal = &sst; var s1-s2;run;
data tx;set tx;group='T';rename  s1=int s2=ind_rc1;run;
data tx;set tx;bl_time=rand('uniform',&uni_lower,&uni_upper);run;
data tx_wide;set tx;id=_n_+&ssp;
z_0=int+rand('normal',0,&error_sd);
if 0.5<bl_time then
z_01=int+(&slope1+ind_rc1)*0.5+rand('normal',0,&error_sd);
     else z_01=.;
if 1.0<bl_time then
z_02=int+(&slope1+ind_rc1)*1.0+rand('normal',0,&error_sd);
     else z_02=.;
z_bl=int+(&slope1+ind_rc1)*bl_time+rand('normal',0,&error_sd);
z_1=int+(&slope1+ind_rc1)*bl_time+1*(&slope1*(1-
&theta)+ind_rc1)+rand('normal',0,&error_sd);
z_2=int+(&slope1+ind_rc1)*bl_time+2*(&slope1*(1-
&theta)+ind_rc1)+rand('normal',0,&error_sd);
z_3=int+(&slope1+ind_rc1)*bl_time+3*(&slope1*(1-
&theta)+ind_rc1)+rand('normal',0,&error_sd);
z_4=int+(&slope1+ind_rc1)*bl_time+4*(&slope1*(1-
&theta)+ind_rc1)+rand('normal',0,&error_sd);
run;
data both;set p_wide tx_wide;run;
data both;set both;bl=z_0;run;

data long_both;set both;
z=z_0;time=0;output;z=z_01;time=0.5;output;
z=z_02;time=1;output;z=z_bl;time=bl_time;output;
z=z_1;time=bl_time+1;output;z=z_2;time=bl_time+2;output;
z=z_3;time=bl_time+3;output;z=z_4;time=bl_time+4;output;
keep id group z time bl_time bl;run;
proc sort data=long_both;by id time;run;

ods output parameterestimates=slope;
proc glm data=long_both;where time<=bl_time;by id;
model z=time/solution;run;
data rc;set slope(rename=(estimate=rc));keep id rc;where
parameter='time';run;
data add;merge long_both rc;by id;run;

proc nlmixed data=add method=firo ;where z ne .;
parms int -0.4673 slope1 -0.11 error_var 0.16 theta 0
v11 0.7793 v21 0.05634 v22 0.04018 ;
```

```
if group='P' then mu=(int+u0)+(slope1+u1)*time;
if group='T' and time<=bl_time then mu=(int+u0)+(slope1+u1)*time;
if group='T' and time>bl_time then mu=(int+u0)+(slope1+u1)*bl_time
+(slope1*(1-theta)+u1)*(time-bl_time);
model z~normal(mu,error_var);
random u0 u1 ~normal([0,0],[v11,v21,v22]) subject=id;
ods output ParameterEstimates =firo;run;
data firo;set firo;iteration=&ite;run;
proc append base=ri.ri_&theta_int&sst data=firo force;run;

data normal;set add;time_new=time-bl_time;where time >=bl_time and z
ne .;run;

proc mixed data=normal noclprint=2;   class id group;
     model z=time_new group*time_new/s;
     random int time_new/sub=id type=un;
     ods output solutionf=s_mixed;run;
data s_mixed;set s_mixed;iteration=&ite;run;
proc append base=ri.mixed_&theta_int&sst data=s_mixed force;run;

proc mixed data=normal noclprint=2;   class id group;
     model z=bl bl*time_new time_new group*time_new/s;
     random int time_new/sub=id type=un;
     ods output solutionf=bl_mixed;run;
data bl_mixed;set bl_mixed;iteration=&ite;run;
proc append base=ri.bl_mixed_&theta_int&sst data=bl_mixed force;run;

proc mixed data=normal noclprint=2;   class id group;
     model z=rc rc*time_new time_new group*time_new/s;
     random int time_new/sub=id type=un;
     ods output solutionf=rc_mixed;run;
data rc_mixed;set rc_mixed;iteration=&ite;run;
proc append base=ri.rc_mixed_&theta_int&sst data=rc_mixed force;run;

%end;/*iteration*/
%mend CRI;

%CRI(sst=200,ssp=200,slope1=-0.09506,uni_lower=0.3,uni_upper=1.2,
error_sd=0.2271563,theta=0,theta_int=0,iteration=1);
```

*======================Scenario 2===

```
/*=============================================~~~~~~~~~~~~~~~~~~~~~~~~~
~~
Simulation based on DIAN data
Two slopes: before Randomization: estimated placebo slope:-0.09506
After randomization: 90% (-0.085554);
```

```
Variance of the slope after randomization:
0.9**2*variance of slope 1 ( 0.0188811);
Correlation between slopes: 0.8
Correlation between int and 2nd slope: 0.4
Assessments: every 0.5 y in RI period, annually in randomization
period
Maximum of 3 assessments in the RI period: 0, 0.5, 1.0
===========================================~~~~~~~~~~~~~~~~~~~~~~~~~
~~*/

data scov_pla(type=COV) ;
   input _TYPE_ $ 1-4 _NAME_ $ 9-10 S1 S2 S3;
   datalines ;
COV     S1      1.0656 0.09253 0.056738
COV     S2      0.09253 0.02331 0.016783
COV     S3      0.056738 0.016783 0.0188811
MEAN            -0.6289 0 0
;run;

%macro
CRI(sst,ssp,slope1,slope2,uni_lower,uni_upper,error_sd,theta,theta_int
,iteration);
%do ite=1 %to &iteration;
*================Pla=====================;
proc simnorm data=scov_pla outsim=pla numreal = &ssp; var s1-s3;run;
data pla;set pla;group='P';rename  s1=int s2=ind_rc1 s3=ind_rc2;run;
data pla;set pla;bl_time=rand('uniform',&uni_lower,&uni_upper);run;
data p_wide;set pla;id=_n_;
z_0=int+rand('normal',0,&error_sd);
if 0.5<bl_time then
z_01=int+(&slope1+ind_rc1)*0.5+rand('normal',0,&error_sd);
     else z_01=.;
if 1.0<bl_time then
z_02=int+(&slope1+ind_rc1)*1.0+rand('normal',0,&error_sd);
     else z_02=.;
z_bl=int+(&slope1+ind_rc1)*bl_time+rand('normal',0,&error_sd);
z_1=int+(&slope1+ind_rc1)*bl_time+1*(&slope2+ind_rc2)+rand('normal',0,
&error_sd);
z_2=int+(&slope1+ind_rc1)*bl_time+2*(&slope2+ind_rc2)+rand('normal',0,
&error_sd);
z_3=int+(&slope1+ind_rc1)*bl_time+3*(&slope2+ind_rc2)+rand('normal',0,
&error_sd);
z_4=int+(&slope1+ind_rc1)*bl_time+4*(&slope2+ind_rc2)+rand('normal',0,
&error_sd);
run;
proc simnorm data=scov_pla outsim=tx numreal = &sst; var s1-s3;run;
data tx;set tx;group='T';rename  s1=int s2=ind_rc1 s3=ind_rc2;run;
data tx;set tx;bl_time=rand('uniform',&uni_lower,&uni_upper);run;
*generate tx data: 4-years after run-in;
data tx_wide;set tx;id=_n_+&ssp;
z_0=int+rand('normal',0,&error_sd);
```

```
if 0.5<bl_time then
z_01=int+(&slope1+ind_rc1)*0.5+rand('normal',0,&error_sd);
     else z_01=.;
if 1.0<bl_time then
z_02=int+(&slope1+ind_rc1)*1.0+rand('normal',0,&error_sd);
     else z_02=.;
z_bl=int+(&slope1+ind_rc1)*bl_time+rand('normal',0,&error_sd);
z_1=int+(&slope1+ind_rc1)*bl_time+1*(&slope2*(1-
&theta)+ind_rc2)+rand('normal',0,&error_sd);
z_2=int+(&slope1+ind_rc1)*bl_time+2*(&slope2*(1-
&theta)+ind_rc2)+rand('normal',0,&error_sd);
z_3=int+(&slope1+ind_rc1)*bl_time+3*(&slope2*(1-
&theta)+ind_rc2)+rand('normal',0,&error_sd);
z_4=int+(&slope1+ind_rc1)*bl_time+4*(&slope2*(1-
&theta)+ind_rc2)+rand('normal',0,&error_sd);
run;
data both;set p_wide tx_wide;run;
data both;set both;bl=z_0;run;

data long_both;set both;
z=z_0;time=0;output;z=z_01;time=0.5;output;
z=z_02;time=1;output;z=z_bl;time=bl_time;output;
z=z_1;time=bl_time+1;output;z=z_2;time=bl_time+2;output;
z=z_3;time=bl_time+3;output;z=z_4;time=bl_time+4;output;
keep id group z time bl_time bl;run;
proc sort data=long_both;by id time;run;

ods output parameterestimates=slope;
proc glm data=long_both;where time<=bl_time;by id;
model z=time/solution;run;
data rc;set slope(rename=(estimate=rc));keep id rc;where
parameter='time';run;
data add;merge long_both rc;by id;run;

proc nlmixed data=add method=firo ;where z ne .;
parms int -0.4673 slope1 -0.11 slope2 -0.10 error_var 0.16 theta 0
v11 0.7793 v21 0.05634 v22 0.04018 v31 0.0637 v32 0.0289296 v33
0.0325458;
if time<=bl_time then mu=(int+u0)+(slope1+u1)*time;
if group='P' and time>bl_time then
mu=(int+u0)+(slope1+u1)*bl_time+(slope2+u2)*(time-bl_time);
if group='T' and time>bl_time then mu=(int+u0)+(slope1+u1)*bl_time
+(slope2*(1-theta)+u2)*(time-bl_time);
model z~normal(mu,error_var);
random u0 u1 u2~normal([0,0,0],[v11,v21,v22,v31,v32,v33]) subject=id;
ods output ParameterEstimates =firo;run;
data firo;set firo;iteration=&ite;run;
proc append base=ri.ri_&theta_int&sst data=firo force;run;
data normal;set add;time_new=time-bl_time;where time >=bl_time and z
ne .;run;

proc mixed data=normal noclprint=2;   class id group;
```

```
     model z= time_new group*time_new/s;
     random int time_new/sub=id type=un;
     ods output solutionf=s_mixed;run;
data s_mixed;set s_mixed;iteration=&ite;run;
proc append base=ri.mixed_&theta_int&sst data=s_mixed force;run;

proc mixed data=normal noclprint=2;   class id group;
     model z= bl bl*time_new time_new group*time_new/s;
     random int time_new/sub=id type=un;
     ods output solutionf=bl_mixed;run;
data bl_mixed;set bl_mixed;iteration=&ite;run;
proc append base=ri.bl_mixed_&theta_int&sst data=bl_mixed force;run;

proc mixed data=normal noclprint=2;   class id group;
     model z=rc rc*time_new time_new group*time_new/s;
     random int time_new/sub=id type=un;
     ods output solutionf=rc_mixed;run;
data rc_mixed;set rc_mixed;iteration=&ite;run;
proc append base=ri.rc_mixed_&theta_int&sst data=rc_mixed force;run;
*/
%end;/*iteration*/
%mend CRI;


%CRI(sst=200,ssp=200,slope1=-0.09506,slope2=-0.085554,
uni_lower=0.3,uni_upper=1.2,error_sd=0.2271563,theta=0,theta_int=0,ite
ration=1);
```
